# The Lack of Light-Dark and Feeding-Fasting Cycles Alters Temporal Events in the Goldfish (*Carassius auratus*) Stress Axis

**DOI:** 10.3390/ani11030669

**Published:** 2021-03-03

**Authors:** Nuria Saiz, Miguel Gómez-Boronat, Nuria De Pedro, María Jesús Delgado, Esther Isorna

**Affiliations:** Fish Neuroendocrinology Group, Department of Genetics, Physiology and Microbiology, Faculty of Biology, Complutense University of Madrid, 28040 Madrid, Spain; nursaiz@ucm.es (N.S.); miguelgomezboronat@ucm.es (M.G.-B.); ndepedro@bio.ucm.es (N.D.P.); mjdelgad@bio.ucm.es (M.J.D.)

**Keywords:** chronobiology, circadian system, animal welfare, fish biology, clock genes

## Abstract

**Simple Summary:**

The circadian system synchronizes physiology and behavior to predictable environmental variations (daily cycles in light, temperature, or food availability). To this purpose, animals possess endogenous oscillators that provide internal temporal signals, thanks to rhythmic clock gene expression. In mammals, disruption of circadian oscillators correlates to overactivation of the stress axis, but this connection is less well known in teleosts. This work aims to learn how the absence of two main external signals (light-dark cycle and feeding schedule) impacts the oscillators in the hypothalamus-pituitary-interrenal) axis of goldfish, studying rhythms in locomotor activity, circulating cortisol, and clock gene expression. The removal of environmental synchronizators caused an increase in basal cortisol levels, indicating stress. Constant darkness also overrode clock gene rhythms in the whole axis but did not impede food anticipatory activity. Feeding at random times disrupted the interrenal clock, but cortisol and activity rhythms remained, concluding that clock gene rhythms in the interrenal tissue are unnecessary for rhythmic cortisol release or diurnality. This work evidences how environmental conditions impact the HPI axis, affecting endocrine and circadian functioning, which need to be considered when progressing towards optimizing fish welfare.

**Abstract:**

Vertebrates possess circadian clocks, driven by transcriptional–translational loops of clock genes, to orchestrate anticipatory physiological adaptations to cyclic environmental changes. This work aims to investigate how the absence of a light-dark cycle and a feeding schedule impacts the oscillators in the hypothalamus-pituitary-interrenal axis of goldfish. Fish were maintained under 12L:12D feeding at ZT 2; 12L:12D feeding at random times; and constant darkness feeding at ZT 2. After 30 days, fish were sampled to measure daily variations in plasma cortisol and clock gene expression in the hypothalamus-pituitary-interrenal (HPI) axis. Clock gene rhythms in the HPI were synchronic in the presence of a light-dark cycle but were lost in its absence, while in randomly fed fish, only the interrenal clock was disrupted. The highest cortisol levels were found in the randomly fed group, suggesting that uncertainty of food availability could be as stressful as the absence of a light-dark cycle. Cortisol daily rhythms seem to depend on central clocks, as a disruption in the adrenal clock did not impede rhythmic cortisol release, although it could sensitize the tissue to stress.

## 1. Introduction

The circadian system has been developed by organisms as an adaptive response to cyclic changes in their environment. This system does not only react to those changes but also anticipates them, modifying physiology and behavior [1].

Circadian oscillators are cell-autonomous and based on transcriptional–translational loops, approximately 24 h long, of the so-called clock genes [2,3,4,5]. The core clock includes transcription-activating factors (encoded by the genes *brain and muscle ARNT-like (bmal)* and *circadian locomotor output cycles kaput (clock)*), which form heterodimers CLOCK:BMAL and bind the regulatory E-box in the promoters of the *period* (*per*) and *cryptochrome* (*cry*) genes, increasing their expression. PER and CRY are transcription-repressing factors, which in turn form heterodimers PER:CRY that inhibit the functioning of CLOCK:BMAL and thereby, their own transcription. In the end, these oscillatory loops conduct the rhythmic expression of multiple clock-controlled genes (CCG), which codify proteins with various physiological functions. These clocks exist in most if not all tissues of fishes, including liver, kidney, skeletal muscle, lung, and the interrenal tissue [5], and specifically, several copies of these clock genes have been cloned in goldfish (*Carassius auratus*), the model species employed in this work.

Circadian clocks need to be “set in time” by environmental factors, called inputs or *zeitgebers* [4,6]. The most documented one in vertebrates is the light-dark cycle, which entrains the suprachiasmatic nucleus in the hypothalamus via the retinohypotalamic tract [1]; but the feeding-fasting cycle is also an important input for the circadian clock, especially in peripheral oscillators, such as the liver [4,6,7,8]. Generally, this could be associated with peripheral oscillators being more dependent on endocrine meal-related responses and gastrointestinal distension signals [9]. Taking into account which *zeitgeber* synchronizes the endogenous oscillator, two main types of clocks have been reported [10]. Light-entrainable oscillators (LEOs) are those in which lighting conditions affect clock gene expression, resetting the rhythms accordingly [5,11]. Oscillators that are mainly synchronized by feeding time are called food-entrainable oscillators (FEOs) [7,12]. While the main LEO appears to be located in the hypothalamus, the anatomic base of the FEO has been elusive [13]. The most widespread effect of FEO synchronization is the food anticipatory activity (FAA), which consists of an increase in locomotion some hours before food is delivered. This behavior pattern is observed under cyclic food availability conditions in all the vertebrates so far studied [12,14], and it persisted several days under food deprivation conditions in mammals [15]. The FAA has been reported in fishes [16], including the goldfish [17,18,19].

In mammals, the suprachiasmatic nucleus has been considered a “master oscillator” [1,11,20] that receives light cues and synchronizes the network of pacemakers distributed in the different tissues [21], although over the last decades, evidence points to a multioscillatory system in many species [2,3,22]. A master clock has not been identified in fishes to date [2,5], which seem to have a less hierarchical circadian system [5]. Another difference with the mammalian circadian system is that teleosts present several copies of clock genes due to genome duplications [23,24]. The autonomy of oscillators in the fish circadian system has been particularly studied in zebrafish (*Danio rerio*), whose peripheral tissues directly respond to light exposure, even when isolated in cell culture [25], a property that has not been proven nor disproven in other fish species to our knowledge.

In this regard, research has been done in order to identify the possible oscillators in goldfish and to determine the role of potential inputs in their entrainment. The goldfish is a well-characterized model in the field of fish physiology, especially in studies of chronobiology [5,26]: A robust FAA even in the absence of a light-dark cycle has been reported [17,18,19]; daily clock gene expression rhythms have been studied in central and peripheral tissues [7,18,27,28,29], and clock genes expression modifications by hormones have also been studied in this teleost [5,24]. The present work is focused on the oscillators located in the hypothalamus-pituitary-interrenal (HPI) axis, which is the regulatory axis of glucocorticoid production in fishes [30]. Both the preoptic hypothalamic nucleus and the lateral tuberal nucleus release neuropeptides, mainly corticotropin-releasing hormone (CRH), that activate the release of pituitary adrenocorticotropic hormone (ACTH). The ACTH flows into the bloodstream and drives the production and secretion of cortisol from the interrenal tissue [31] that consists of steroidogenic cells embedded in the head kidney in most teleost fish [32,33]. Glucocorticoids mediate the main endocrine stress response in vertebrates, stimulating catabolism of carbohydrates, protein, and lipids and raise blood glucose [34].

In addition to the stress response, the HPI axis takes part in the circadian system. Like melatonin, glucocorticoids are considered one of the main hormonal outputs of the circadian system. At least in mammals, under unstressed circumstances, glucocorticoid levels are low during the periods of sleep or inactivity and increase before waking, peaking in the morning on diurnal animals [35]. Daily cortisol rhythms are less robust in teleosts and depend on several factors (ontogeny, salinity, photoperiod, feeding regime) and species [5,36]. In goldfish, circulating cortisol showed daily rhythms in animals maintained under 12L:12D photoperiod and fed every day at mid-photo phase [7]. Such rhythms were accomplished by diel oscillations of gene expression in the three tissues that are part of the axis: hypothalamus, pituitary, and interrenal tissue in goldfish [7,27] and the homologs of several other vertebrates [37,38,39,40].

From the study of the literature of the field, it is likely that a temporal homeostasis of the HPI axis could modulate stress responses in fishes. Thus, this work aimed to determine a possible crosstalk between the circadian system and HPI axis. To this end, we have studied the effects of the light-dark cycle and feeding schedule removal on the entrainment of daily rhythms in locomotor activity, circulating cortisol levels, and clock gene expression in the goldfish HPI oscillators.

## 2. Materials and Methods

### 2.1. Animals and Housing

Goldfish (14 ± 4 g body weight, bw) were acquired from a local commercial supplier (ICA, Madrid, Spain) and kept in 60-L tanks with filtered and aerated fresh water (21 ± 2 °C), in groups of 9–10 individuals. Standard maintenance conditions were at 12L:12D photoperiod (lights-on at 8 a.m., equivalent to *zeitgeber* time 0, ZT 0), and daily feeding at ZT 2 with food pellets (1% bw; Sera Pond Biogranulat, Heinsberg, Germany) by automatic feeders. Acclimation of animals was carried out for 3 weeks before the beginning of the experiment. The experiment complied with the Guidelines of the European Union Council (UE63/2010) and the Spanish Government (RD53/2013) for the use of animals in scientific proposals and was approved by the Animal Experimentation Committee of the Complutense University (O.H.-UCM-25-2014) and the Community of Madrid (PROEX 107/14).

### 2.2. Experimental Design

Animals (N = 147) were divided into 15 tanks (60 L, with a biomass of 136.8 ± 1.48 g (mean ± SEM) and 3 experimental groups, *n* = 49 (5 tanks/experimental group). All groups were fed once per day with automatic feeders (1% bw) with different photoperiodic conditions and feeding time. (1) LD-2 group: fish were left under light–dark cycle (12L:12D photoperiod, lights on at ZT 0) and daily fed at ZT 2 (standard conditions). (2) LD-R group: fish remained under the same photoperiod (12L:12D, lights on at ZT 0), and food was provided on a random schedule (i.e., at a different time each day, provided by random number generator software—RAND function—of Microsoft Excel^®^). (3) DD-2 group: fish kept under constant darkness (24 h, DD) and daily fed at circadian time 2 (CT 2). The whole experiment was performed simultaneously using two different facilities (one with 12L:12D photoperiod and the other with constant dark).

After 30 days under these conditions, goldfish were sampled every 4 h through a 24 h cycle (*n* = 7/sampling point) at ZT 3, ZT 7, ZT 11, ZT 15, ZT 19, ZT 23, and ZT 3b (ZT 3 of the next day), taking fish from more than one aquaria in every sampling point to avoid tank bias. Food was given as scheduled (ZT 2/CT 2) the first day of sampling but not the second day before the last sampling point (ZT 3b). Blood was collected from the caudal vein of anesthetized animals (tricaine methane sulfonate, MS-222, 0.14 g/l; Sigma–Aldrich, St. Louis, MO, USA), and plasma was stored at −80 °C until assay. Fish were then sacrificed by anesthetic overdose (MS-222, 0.28 g/L; Sigma–Aldrich, St. Louis, USA), and hypothalamus, pituitary, and head kidney were extracted, frozen in liquid nitrogen, and stored at −80 °C until analysis.

### 2.3. Locomotor Activity Recordings

Locomotor activity was recorded throughout the 30 days of the experiment using an actimeter composed of a group of infrared photocells (Omron Corporation E3S-AD12, Japan) fixed onto aquarium walls and data acquiring software (Adq16, Micronec, Madrid, Spain). In each tank, two photocells were placed below the automatic feeder (for recording feeding-related activity) and four photocells at a height of 3–9 cm above the bottom (for recording general locomotor activity). Aquaria were covered with opaque paper to minimize external interferences during the experiment. The number of light beam interruptions was automatically registered every 10 min. All registered data were analyzed using El Temps^®^ (Prof. Antoni Díez Noguera, Univ. of Barcelona, Spain) to obtain profiles of average daily activity rhythms, actograms, and periodograms from all the experimental groups.

### 2.4. Cortisol Plasmatic Levels

Plasma cortisol levels were determined by enzyme-linked immunosorbent assay (ELISA) using a commercial kit (Demeditec, Schleswig-Holstein, Germany), previously validated for goldfish plasma [7,41]. The lowest analytical detectable level of cortisol that can be distinguished from the zero calibrator was 3.79 ng/mL. Free cortisol values were expected to be within the range described by the manufacturer (10–800 ng/mL). Therefore, no dilution was necessary. All samples were measured in duplicate.

### 2.5. Gene Expression Analysis

Total RNA from hypothalamus, pituitary, and head kidney was isolated using TRI^®^ Reagent (Sigma–Aldrich, St. Louis, USA) and treated with RQ1 RNase-Free DNase (Promega, Madison, WI, USA). Then, 0.3 µg of total RNA was reverse transcribed into cDNA in 25 μL reaction volume using random primers (Invitrogen, Waltham, MA, USA), RNase inhibitor (Promega, Madison, USA), and SuperScript II Reverse Transcriptase (Invitrogen, Waltham, USA). RT-qPCR was carried in each sample in duplicate in a CFX96TM Real-Time System (Bio-Rad Laboratories, Hercules, CA, USA), using iTaqTM Universal SYBR Green Supermix (Bio-Rad Laboratories Hercules, USA), into a 96-well plastic plate loaded with 1 μL of cDNA and 0.5 μL of forward and reverse primers 10 µM, to a final volume of 10 μL. Each PCR plate also included a standard dilutions curve to ensure the efficiency of PCR reactions (90–105%) and water and pre-RT RNA as negative controls.

The RT-qPCR protocol included an initial denaturation step of 95 °C for 30 s, followed by 40 cycles of a two-step amplification program (95 °C for 5 s and 60 °C for 30 s). A melting curve was systematically monitored (temperature gradient at 0.5 °C/5 s from 70 to 90 °C) to verify reaction specificity. Gen Data Bank reference numbers and primers (Sigma–Aldrich, St. Louis, USA) sequences utilized for target genes and the reference gene are shown in Supplementary Materials Table S1. To determine relative mRNA expression (fold change), we used the 2^−ΔΔCt^ method [42] and normalized it to the group with the lowest expression.

### 2.6. Data Analysis

Statistical differences in plasma cortisol levels among groups and hours were compared by a two-way ANOVA (factors: experimental group and sampling time). The presence of 24-h rhythms in plasma cortisol was determined by Cosinor analysis, which reports the adjustment of the data to sinusoidal functions by the least-squares method [43], using the formula f(t) = M + A × cos(t × π/12 − Φ), where f(t) is level of gene expression at a given time, the mesor (M) is a rhythm-adjusted mean, A is the amplitude of the rhythm, t is time in hours, and Φ is the acrophase or time of maximum expression. Nonlinear regression allowed the estimation of M, A, Φ, and their standard error (SE), which were calculated on the residual sum of squares in the least-squares fit [43,44]. The significance of COSINOR analysis was determined by the noise/signal of amplitude calculated from the ratio SEM(A)/A [28]. Data were considered to display a daily rhythm if had *p* < 0.05 by ANOVA, and SEM(A)/A < 0.3 by Cosinor analysis.

With respect to the locomotor activity average profiles, the rhythm acrophases were calculated by Cosinor analysis, and significance was evaluated via the Rayleigh test. The rhythm period was calculated by Sokolove–Bushell periodograms. All significance thresholds were set at *p* < 0.05 (El Temps^®^).

To compare gene expression data of different sampling points (i.e., times of day), we used one-way ANOVA followed by a post-hoc multiple comparison test (Student–Newman–Keuls, SNK), using SigmaPlot 12.0 (samples of each experimental group were measured in different plates; thus values were relativized in each group and a two way ANOVA could not be performed). When necessary to fulfill normality or homoscedasticity criteria, data were transformed to logarithmic or square root scale. In all tests performed, we considered a *p* < 0.05 statistically significant. The presence of 24-h rhythms in gene expression was determined by cosinor analysis as above explained.

## 3. Results

### 3.1. Locomotor Activity Recordings

**Figure 1:**
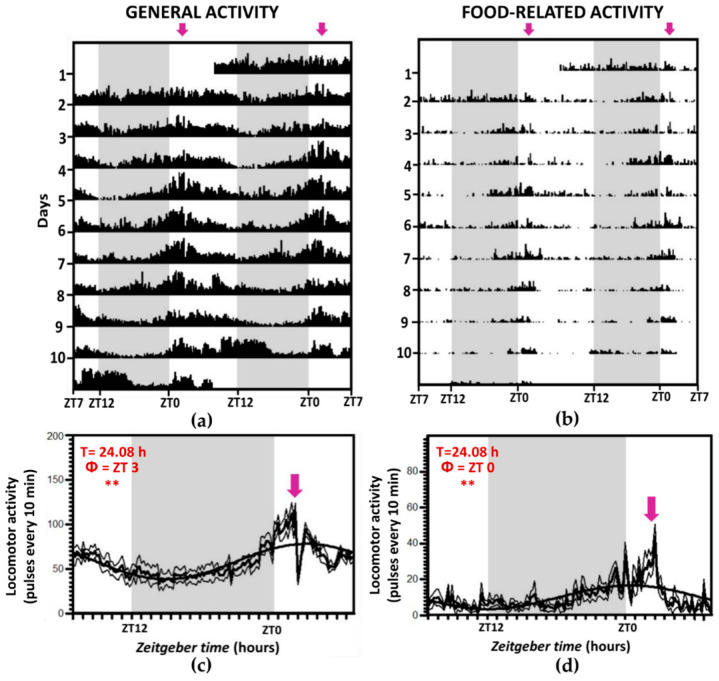
Locomotor activity (general (**a**,**c**) and food-related (**b**,**d**)) for group LD-2, with a 12L:12D photoperiod and fed at ZT 2. The shaded zones indicate the darkness period, and the arrows point to the feeding time. (**a**,**b**): double-plot actograms, in which the X-axis represents the time of the day of two consecutive days, and the Y-axis represents the days on which activity was registered. (**c**,**d**): Average waveform of locomotor activity (same data). Data are expressed as mean ± SEM. Acrophase (Փ) and significance of the Rayleigh test for a 24-h rhythm are shown ** *p* < 0.01. T = rhythm period (when significant) according to Sokolove–Bushell periodograms.

**Figure 2:**
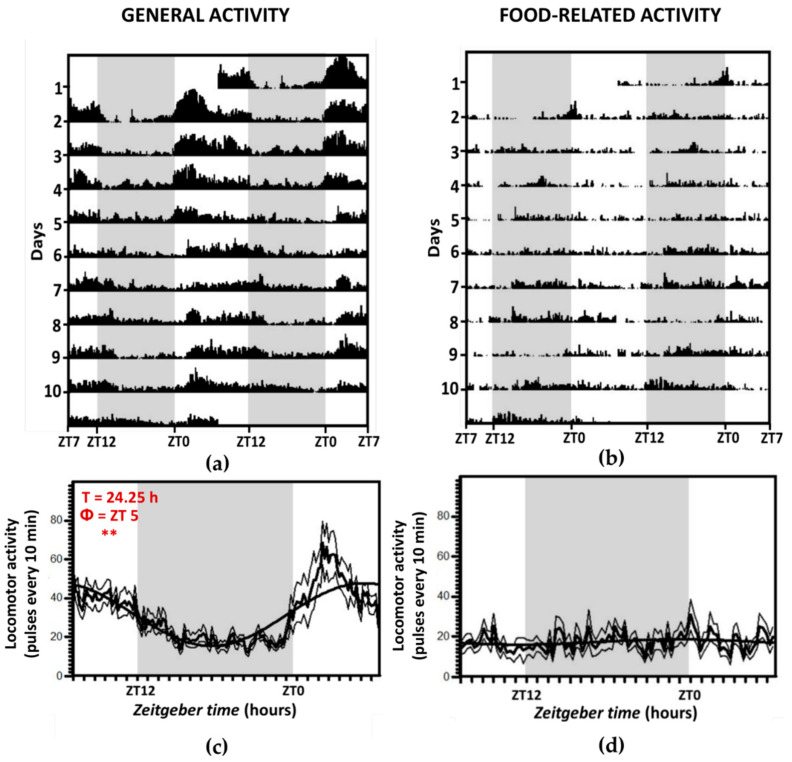
Locomotor activity (general (**a**,**c**) and food-related (**b**,**d**)) for group LD-R, with a 12L:12D photoperiod and fed at random times. The shaded zones indicate the darkness period. (**a**,**b**): double-plot actograms, the X-axis represents the time of day of two consecutive days, and the Y-axis represents the days on which activity was registered. (**c**,**d**): Average waveform of locomotor activity. Data are expressed as mean ± SEM. Acrophase (Փ) and significance of the Rayleigh test for a 24-h rhythm are shown ** *p* < 0.01. T is the period (when the rhythm is significant) according to Sokolove–Bushell periodograms.

**Figure 3:**
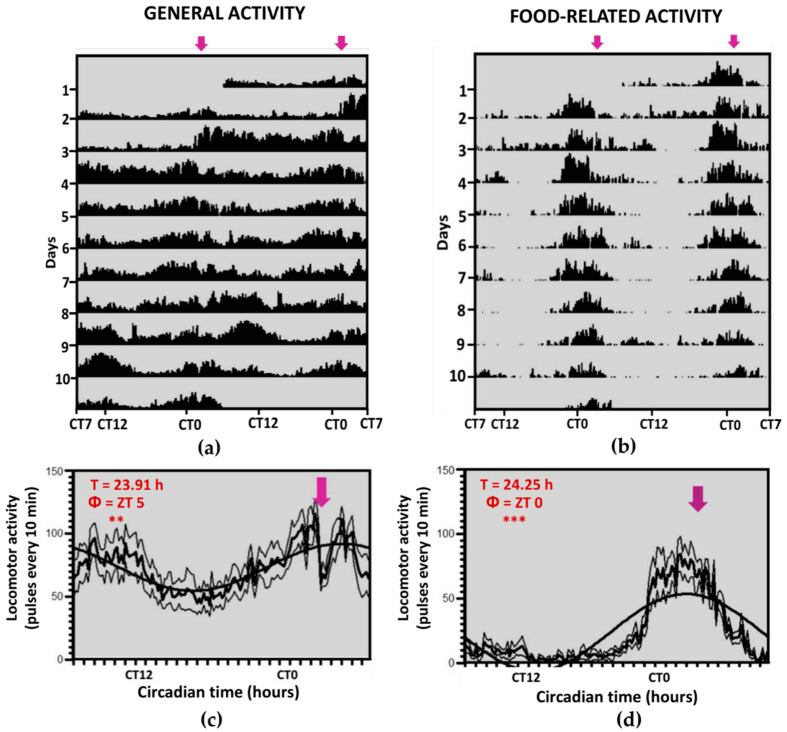
Locomotor activity (general (**a**,**c**) and food-related (**b**,**d**)) for group DD-2, under constant darkness and fixed feeding. The shaded zones indicate the darkness period. (**a**,**b**): double-plot actograms, in which the X-axis represents the time of the day of two consecutive days, and the Y-axis represents the days on which activity was registered. (**c**,**d**): Average waveform of locomotor activity (same data). Data are expressed as mean ± SEM. Acrophase (Փ) and significance of the Rayleigh test for a 24-h rhythm are shown ** *p* < 0.01, *** *p* < 0.001. T = rhythm period (when significant) according to Sokolove–Bushell periodograms.

Representative actograms and daily profiles of locomotor activity in each experimental group are shown in Figure 1, Figure 2 and Figure 3, showing separately general activity (Figure 1a, Figure 2a and Figure 3a) and food-related activity (Figure 1b, Figure 2b and Figure 3b). Fish maintained under a 12L:12D photoperiod and fed at 10:00 h (ZT 2, LD-2 group) displayed a locomotor activity rhythm of ~24 h in both general (Figure 1a) and food-related (Figure 1b) activity. The activity was higher during the light period; 57% of the pulses in the tank were registered during the photophase vs. 43% during the scotophase. Moreover, animals were also synchronized to the feeding schedule, as observed in FAA actograms (pronounced increase in activity just before mealtime), as 21% of the total activity in the tank happened during the 3 h preceding mealtime (Figure 1c,d).

Locomotor activity of fish maintained under 12L:12D and randomly fed (LD-R group) is shown in Figure 2. As it can be observed, fish maintain a diurnal activity pattern, with higher differences in daytime activity levels compared to nighttime activity than observed in the control group (LD-2), i.e., 65% of pulses were registered during the photo phase, and the acrophase was at ZT 5), displaying a rhythmic diurnal behavior. The lack of a daily feeding schedule resulted in uniform levels of activity near the automatic feeders along the 24 h (Figure 2b,d), and food-associated rhythmic activity disappeared (only 11% of pulses occurred at 7–10 a.m. interval).

Fish under 24D and scheduled feeding (DD-2 group) showed higher general activity during subjective daytime than nighttime (56% vs. 44%; Figure 3a), leading to rhythmic 24-h oscillations, with an acrophase at ZT 5, that are possibly driven by scheduled feeding. Moreover, there were peaks of activity that seem to anticipate the time of feeding, sustaining a significant rhythm of 24 h, and levels of FAA like those from the LD-2 group (22%).

### 3.2. Daily Variations of Clock Gene Expression

**Figure 4:**
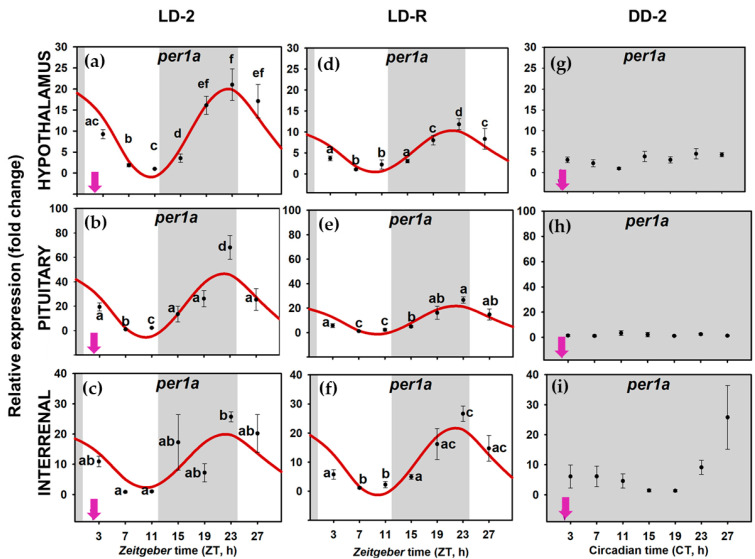
Relative expression of period 1a (per1a) in hypothalamus (**a**,**d**,**g**), pituitary (**b**,**e**,**h**) and interrenal tissue (**c**,**f**,**i**) of goldfish. Data are expressed as mean ± standard error (n = 8). Grey areas indicate the darkness period, and arrows point to the feeding time when it was scheduled (*zeitgeber* time 2 (ZT 2). Different letters show significant differences among groups (ANOVA and Student–Newman–Keuls (SNK) test). The curves show sinusoidal periodic functions obtained by cosinor analysis when it was significant (noise/signal < 0.3).

**Figure 5:**
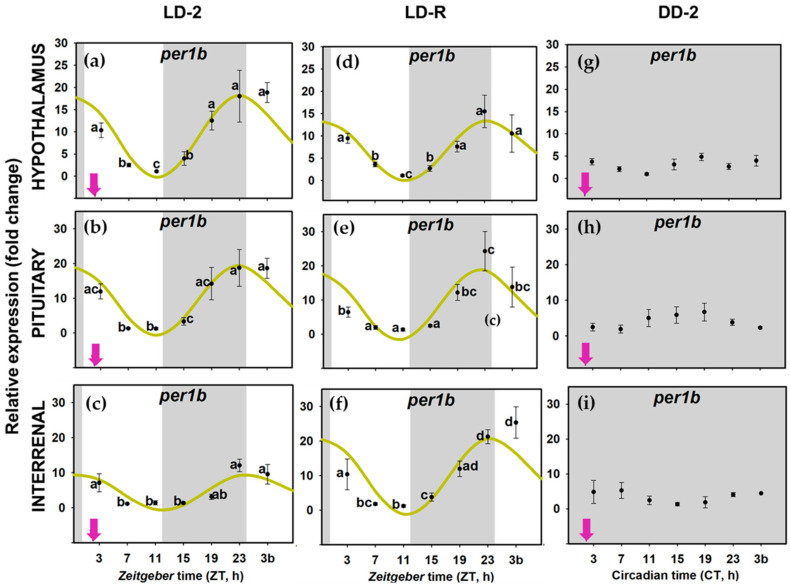
Relative expression of *per1b* in hypothalamus (**a**,**d**,**g**), pituitary (**b**,**e**,**h**) and interrenal tissue (**c**,**f**,**i**) of goldfish. Data are expressed as mean ± standard error (*n* = 8). Grey areas indicate the darkness period, and arrows point to the feeding time when it was scheduled (ZT 2). Different letters show significant differences among groups (ANOVA and SNK test). The curves show sinusoidal periodic functions obtained by cosinor analysis when it was significant (noise/signal < 0.3).

**Figure 6:**
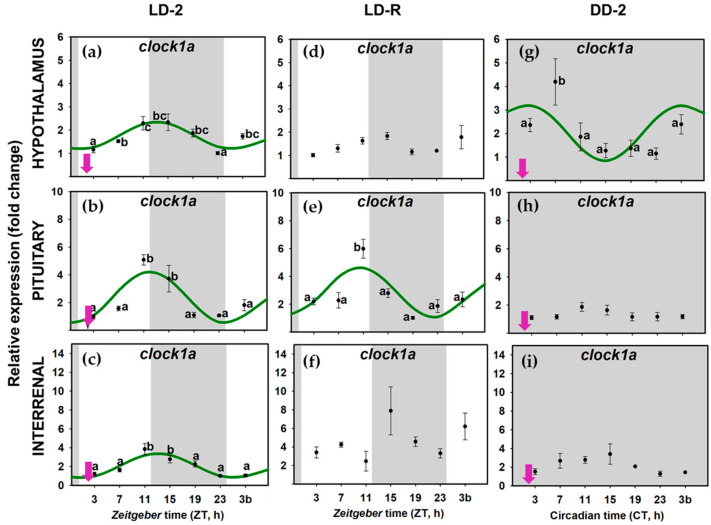
Relative expression of *clock1a* in hypothalamus (**a**,**d**,**g**), pituitary (**b**,**e**,**h**) and interrenal tissue (**c**,**f**,**i**) of goldfish. Data are expressed as mean ± standard error (*n* = 8). Grey areas indicate the darkness period, and arrows point to the feeding time when it was fixed. Different letters show significant differences among groups (ANOVA and SNK test). The curves show sinusoidal periodic functions obtained by cosinor analysis when it was significant (noise/signal < 0.3).

**Figure 7:**
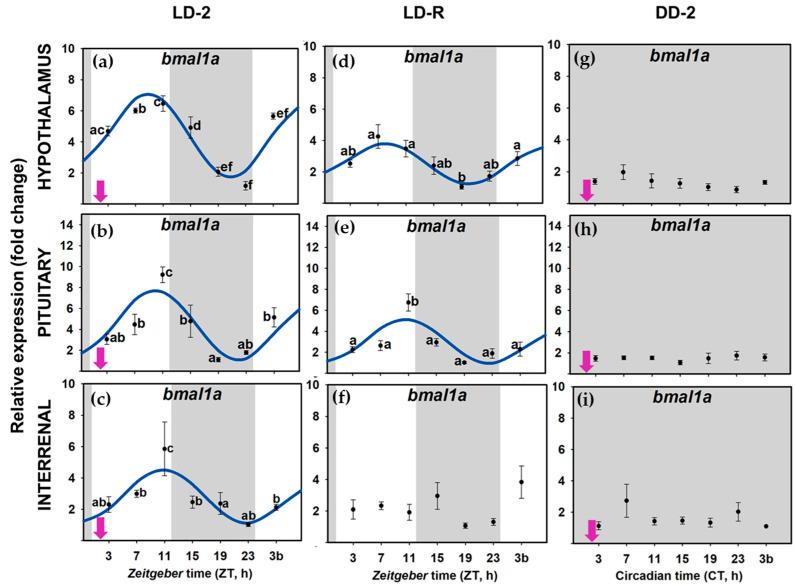
Relative expression of *bmal1a* in hypothalamus (**a**,**d**,**g**), pituitary (**b**,**e**,**h**) and interrenal tissue (**c**,**f**,**i**) of goldfish. Data are expressed as mean ± standard error (*n* = 8). Grey areas indicate the darkness period, and arrows point to the feeding time when it was scheduled (ZT 2). Different letters show significant differences among groups (ANOVA and SNK test). The curves show sinusoidal periodic functions obtained by cosinor analysis when it was significant (noise/signal < 0.3).

When food availability and light-dark were cyclic LD-2 group), all the examined clock genes (*per1a*, *per1b*, *clock1a*, and *bmal1a*) presented daily rhythms of expression in the three tissues of the HPI axis, and daily oscillations were synchronic (similar acrophases) in all the genes at the whole axis (Figure 4a–c,Figure 5a–c, Figure 6a–c and Figure 7a–c); Table S2). The rhythm of *per1a* expression displayed pronounced fluctuations in all the tissues (20–40-fold, Figure 4a–c), while *per1b* behaved almost identically to its paralog, but with lower amplitudes (10–20-fold, Figure 5a–c). The *bmal1a* and *clock1a* rhythms were less marked, with amplitudes lower than 10, especially reduced in the interrenal tissue (Figure 6a–c and Figure 7a–c). Moreover, the genes from the positive half of the loop (*clock1a* and *bmal1a*) had acrophases or peaks of expression at the end of the light phase 89u ≈ ZT 11 h), that were in antiphase with those from the elements of the negative arm of the loop (*per1a* and *per1b*, Φ ≈ ZT 23 h), which peaked at 1 h before the light onset (Figure S1, Table S2).

However, when food was provided on a random schedule (LD-R group), several components of the molecular machinery of the circadian clock were negatively affected. The daily expression of *clock1a* remained rhythmic in the hypophysis (Figure 7d–f) but not in the hypothalamus or the interrenal tissue (Figure 7d–f). *Bmal1a* reduced its amplitude in the hypothalamus and hypophysis (compared to LD-2, Table S2) and lost its rhythmicity in the interrenal tissue (Figure 6d–f). The expression of *per1* genes kept daily rhythms with similar acrophases in the three tissues (Figure 4d–f and Figure 5d–f), but *per1a* underwent a nearly half-fold decrease in the amplitude of the rhythm in the hypothalamus and pituitary (Table S2). Surprisingly, the amplitude of *per1b* in the interrenal tissue doubled in fish under LD-R (Table S2).

In the DD-2 group, in the absence of a light–darkness cycle, the molecular pacemaker became substantially impaired despite fish having had a scheduled mealtime. All studied clock genes in the hypothalamus (Figure 4g, Figure 5g, Figure 6g and Figure 7g), pituitary (Figure 4h, Figure 5h, Figure 6h and Figure 7h), interrenal tissue (Figure 4i, Figure 5i, Figure 6i and Figure 7i) lost their rhythm of expression, except for *clock1a* in the hypothalamus (Figure 7g), which exhibited a significant daily rhythm with a displaced acrophase around ZT 7 (compared to ZT 13 in LD-2 group Table S2).

### 3.3. Daily Cortisol Variations

**Figure 8:**
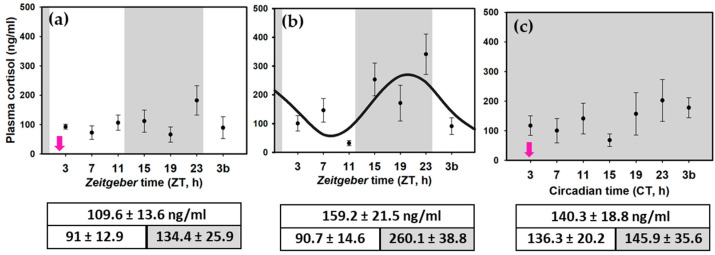
Daily profiles of plasma cortisol in LD-SF2 (**a**), LD-RF (**b**), and DD-SF2 (**c**) goldfish. Data are expressed as mean ± standard error (*n* = 8). Grey areas indicate the darkness period, and arrows point to the feeding time when it was scheduled (ZT 2 or CT 2). The two-way ANOVA was significant, showing differences among sampling points (*p* < 0.05) but not an interaction between sampling points and experimental group (*p* = 0.056). The post-hoc test indicated significant differences between ZT 23 (or CT 23 in the DD group) and ZT(CT) 11, and between ZT(CT) 23 and ZT(CT) 7. The curve (**b**) shows the sinusoidal periodic function obtained by cosinor analysis when it was significant (noise/signal < 0.3). The tables below the figures show the cortisol (ng/mL) (mean ± SEM) through the 24 h cycle, during the photophase and the scotophase (lower panel; subjective day and subjective night in 4c) in each experimental group.

The mean value of cortisol levels during 24-h was lower in animals under 12L:12D fed at ZT 2 (109.6 ± 13.6 ng/mL; Figure 8) than in fish under constant dark fed at ZT 2 (140.3 ± 18.8 ng/mL; Figure 8). The highest cortisol mean levels were found in the randomly fed group (159.2 ± 21.5 ng/mL; Figure 8), even if the LD cycle was present. Such higher levels of cortisol result from the higher nocturnal values, as diurnal circulating cortisol was similar to that found in LD-2 fish. Altogether, these results suggest that the absence of just one of the two *zeitgebers* is a stressor for these animals. Daily profiles of plasma cortisol showed a rise in cortisol before the light onset (or the subjective light in the DD-2 group, Figure 8c) in all the groups. The two-way ANOVA test indicated significant differences between ZT 23 (CT 23 in the DD group) and ZT 11 (CT 11) and ZT 23 and ZT 7 (CT 7). The interaction between factors (sampling time and experimental group) was in the limit of significance (*p* = 0.056), suggesting that although statistics cannot confirm it, the nocturnal increase in cortisol was not homogenous in the different groups. It seems that a peak of cortisol occurred at ZT 23 in the LD-2 group, being maintained at low levels during the rest of the day (Figure 8a), while the day-night variations diminish in constant dark (Figure 8c). In fish randomly fed under an LD cycle (LD-R), cortisol was maintained at high levels during the night (Figure 8b), and cosinor analysis indicated a significant sinusoidal rhythm. Such different daily profiles can be evidenced when comparing the diurnal vs. nocturnal cortisol. Thus, diurnal values in LD-2 and LD-R were similar (91 and 90.7; Figure 8), although nocturnal values were two-fold higher in randomly fed fishes (134.4 vs. 260.1; Figure 8). Fish maintained under constant dark presented homogeneous cortisol levels through the day (diurnal mean 136.3 vs. nocturnal mean 145.9 ng/mL; Figure 8).

## 4. Discussion

The presented data highlight the relevance of scheduled food availability as a potent synchronizer of locomotor activity in goldfish while supporting the previously reported diurnal behavior of this species. Results also showed that the light-dark cycle is necessary to maintain functional oscillators in the HPI axis, although the interrenal clock is also altered in randomly fed fish. Interestingly, disrupted clock gene oscillations in the interrenal tissue do not impede cortisol rhythms, although it could alter stress response (in terms of cortisol values) in goldfish.

Results evidenced the strong role of scheduled feeding in locomotor activity since in animals maintained in constant darkness, a scheduled feeding evoked a strong FAA, which was not subservient to daily cortisol rhythms or clock gene expression rhythms in any of the studied oscillators of HPI axis, neither in central (hypothalamus) nor in peripheral (pituitary, interrenal tissue) locations.

By contrast, daily cortisol rhythms, as well as general locomotor activity (not related to food intake), seem to be dependent on an LEO probably located at the central level, as disrupted clock gene expression rhythms in the interrenal tissue do not prevent cortisol and locomotor activity rhythms when an LD cycle is present, but such rhythms are dampened in constant darkness.

Goldfish showed significant daily locomotor activity rhythms with periods of ~24 h in the presence of both *zeitgebers*, photoperiod, and mealtime, and in the presence of either of them alone. The fact that locomotor activity rhythms were maintained even when the LD cycle was removed agrees with the hypothesis that daily activity rhythms in goldfish are driven by an endogenous circadian oscillator entrained by light (an LEO). If such rhythmic behavior is only a passive consequence of environmental cycles or it is driven by an endogenous clock cannot be concluded from our results, as both, or at least one *zeitgeber* (24 h light/dark or feeding/fasting cycles) were always present. Thus, it cannot be ruled out that the observed locomotor activity rhythms are a masking effect of these *zeitgebers* [45]. However, many previous studies indicate that endogenous oscillators exist in goldfish, and locomotor activity rhythms in this teleost are driven by both LEOs and FEOs [7,18,46,47].

In the three experimental groups, most of the locomotor activity occurred during the photophase (when an LD cycle was present) or the subjective daytime (under continuous darkness), supporting that goldfish are preferably diurnal, as is generally accepted for this species [48,49], even a interindividual variance also exists [46]. Remarkably, animals kept under an LD cycle but randomly fed (LD-R group) showed higher diurnality (% of total activity shown during the photophase) than fish with both *zeitgebers* (65% vs. 57%). This difference is not a possible artifact caused by feeding during the photophase because the meals in randomly fed fish were often offered during the nighttime. The higher intensification of activity during the photophase in these LD-R fish could be due to the absence of a fixed feeding time, which might provoke the circadian system to be more reliant on light–dark cycles. It could be noted that surprisingly daily cortisol rhythms were also clearer in fish randomly fed under a 12L:12D photocycle than in fish with scheduled feeding, as will be discussed below.

According to previous results, the preference for activity during the light phase of the daily photocycle in goldfish is not dependent on feeding time since it is maintained in fish acclimated to eating during the night and displaying FAA in advance [7,46,50]. Altogether these results support a certain independence in the mechanisms underlying both feeding-associated and general locomotor activities, i.e., the FEO and the LEO.

As expected, the FAA was apparent in goldfish fed daily at ZT 2 (LD-2 and DD-2 groups) a few hours before mealtime, as in most cases of scheduled feeding in teleosts [7,18,50], reinforcing the presence of a functional FEO regulating locomotor activity.

Complementary to this, feeding-associated rhythms (such as the FAA) were absent in randomly fed animals (LD-R), as the unpredictability of their mealtime did not allow the entrainment of the FEO, in agreement with previous results in this teleost [18,47], in sea bream (*Sparus aurata)* [50,51] and zebrafish [16]. The acrophase of daily locomotor activity rhythms in fish maintained in constant darkness seems to be centered around feeding time. In addition, the presence of FAA in the absence of a photoperiod confirms that this kind of activity is independent of the light/dark cycle, similar to previous results under constant light in zebrafish [16], turbot (*Scopthtalmus maximus*) [52], and goldfish [18]. However, these results contrast with a recent report in turbot, where the FAA under constant darkness and scheduled feeding was not observed [52]. Few studies have reported FAA under constant darkness in fishes, in zebrafish, and in cavefish (*Hreatichbthys andruzzii*), a fish that naturally lives in dark environments [53]. These results are indicative of light-independent circadian entrainment and add further weight to the role of FEOs in temporal homeostasis in teleosts.

Many signals that are associated with feeding could be mediating entrainment by mealtimes, such as sensory stimuli, feeding-related activity, gastrointestinal distension, and motility, nutrient metabolism, absorption, or postprandially released hormones as insulin [9,54]. Other hormones, such as glucocorticoids, could also be of special importance. In mammals, glucocorticoids are related to locomotor activity in a bidirectional way; they anticipate the onset of activity [55], and activity by itself synchronizes central and peripheral clocks, possibly involving glucocorticoid release [56]. Although they cannot be considered feeding-related hormones, such as insulin, glucocorticoids regulate energy storage and are potent internal temporal messengers for peripheral tissues in mammals [4,20,55] and teleosts [5], since they reset circadian oscillators probably by inducing the clock gene *per1a* [24,53]. In addition, the daily rhythms in circulating cortisol and clock genes in interrenal tissue are disrupted in goldfish fed in the middle of the night [7]. Thus, it is tempting to hypothesize that cortisol in fish could be involved in FAA by synchronizing locomotor activity with clock gene oscillations in endogenous clocks. However, the present data show a pronounced FAA in animals maintained in constant darkness (and fed at a fixed time), while cortisol fluctuation through the day was minimal, and clocks located in the hypothalamus, pituitary, and interrenal tissue did not seem to be functional in these fish (as most of the clock genes were not expressed rhythmically in the HPI axis under such conditions). Thus, as will be discussed below, the FAA could be independent of both rhythmic oscillations of clock genes in the HPI axis and plasma cortisol levels, or the FEO could be located somewhere else. To date, the signal(s) that entrains the FEO generating the FAA is unknown, and it is likely to be redundantly mediated by a combination of exogenous and endogenous factors [4,45,57].

Considering plasma cortisol as an indicator of a stressful condition, it is evident that animals are less stressed under the presence of both daily photocycle and feeding schedule. Cortisol increased in fish under constant dark fed at ZT 2, while the highest cortisol levels happen in randomly fed fish, even if an LD cycle was present. This suggests that the absence of only one of the two *zeitgebers* is a stressor for these animals, the lack of a feeding schedule being even worse than losing the LD cycle. In sea bream (a diurnal species), postprandial cortisol increment was smaller in fish fed at midday than in animals fed in the middle of the scotophase, suggesting that an optimal feeding schedule could decrease cortisol and thus increase welfare [58].

It is well established that mean plasma cortisol levels are not the only parameter to be considered when studying the HPI function in mammals; temporal events in the HPI axis are also important [5,59]. Thus, the relevance of a robust rhythm of cortisol, with high nocturnal levels (as occurs in randomly fed fish) versus a dampened rhythm with lower cortisol levels (in LD-2 and DD-2 groups) should be investigated. Although daily cortisol rhythms might not be a rule in all teleosts [36], daily cortisol rhythms have been described in several fish species, including sea bream, permit (*Trachinotus falcatus*), or goldfish [5,7,58,60]. Regarding cortisol rhythms, the most striking result of the present work is the apparent independence of daily variations of cortisol and interrenal clock gene oscillations. Nevertheless, a relationship with the hypothalamic clock cannot be discarded, as discussed below.

When goldfish were maintained under 12L:12D photoperiod and fed at ZT 2 (LD-2 group), in the three targets studied in the HPI axis (hypothalamus, pituitary, and interrenal tissue), *per1a* and *per1b* expression rhythms were in antiphase with *clock* and *bmal1a* (around a 12-h lag), pointing to a functional molecular clock. In addition, in this group, clock gene expression cycles were synchronic in the three tissues, with simultaneous acrophases for each studied gene (Figure 5, Table S2). *Bmal1a* and *clock1a* expression peaked during the second half of the photo phase (*clock1a*~ZT 12–13 h; *bmal1a*~ZT 8–11 h), while *per* transcripts increased at the end of the scotophase (*per1a*~ZT 22 h; *per1b*~ZT 23–24 h), as is a general rule in diurnal fish [5,61], including goldfish clocks [7,29,46]. Therefore, the data revealed that molecular clocks in the three components of the HPI axis—hypothalamus, pituitary, and interrenal tissue of goldfish—work in phase. When analyzing rhythm parameters, it must be considered that there is a time lag between the peak in mRNA (measured in this work) and protein concentration, which has been estimated to be around 6 h for *per1* [11]. It is generally accepted that a similar time-lag between mRNA and protein levels would occur for other clock genes. Thus, it is expected that a 12-h shift in acrophases of clock gene expression would be followed by a 12-h shift in the peaks for their respective proteins. However, posttranslational regulators that would modify such acrophases might also occur [62,63].

Our results support that the functioning of oscillators in the HPI axis of goldfish is dependent on the light/dark cycle, as the daily rhythms of clock genes were abolished in the absence of this *zeitgeber* (under DD). The only exception was *clock1a* that showed significant rhythms in the hypothalamus of fish under constant dark and scheduled feeding, but this rhythm was lost in LD-R group, which could suggest that this gene has a link to feeding time in this tissue. These results agree with the widely accepted role of the hypothalamus as a LEO. In fact, acrophases of clock genes rhythms in the hypothalamus are only slightly affected by a 12-h shift in feeding schedule in goldfish [7]. In support of the importance of the LD cycle for hypothalamic clock synchronization in goldfish, it is reported that under constant light conditions (LL), *per1a* and *per3* slightly maintained rhythm if feeding time was not modified, but other clock gene rhythms were impaired (*per2a, cry1-3*) [18]. In these goldfish maintained under constant light, a 12 h-shift in feeding schedule and random feeding impaired *per1a* rhythm in the hypothalamus, suggesting that the *per1a* rhythm observed in LL was previously established but, in the absence of an LD cycle, it cannot be resynchronized to a new feeding schedule [18]. Lastly, we cannot discard that rhythms in discrete hypothalamic nuclei could occur, as the hypothalamus is a heterogeneous brain region with many different nuclei. In fact, the dorsomedial hypothalamus has been proposed as a central FEO in rodents since it expresses *per1* rhythmically only under restricted feeding, and it can inhibit FAA when it does not oscillate properly, unlike other hypothalamic nuclei [64,65,66].

Surprisingly, not only the hypothalamus but also the pituitary and the interrenal tissue seem to act as LEOs in goldfish because daily rhythms of clock genes in these tissues were abolished in constant darkness. The pituitary in goldfish behaves similarly to the hypothalamus since the rhythms of clock genes in the pituitary were unaffected by a 12-h shift of feeding time [7], and a random feeding schedule had little to no effect on its oscillation (present results). However, this is not exactly the case for the interrenal tissue. The positive limb of the interrenal clock (*bmal1a* and *clock1a* genes) lost its rhythmicity under random feeding conditions, while the *per* genes were unaffected. This result differs from a previous report that showed *per1* genes precede feeding time, suggesting that the interrenal tissue in goldfish may behave as a FEO [7]. According to the present results, the interrenal tissue differed from other peripheral clocks in goldfish, such as the liver and the gut, which can be entrained by the feeding schedule under constant light or darkness [18,46]. The interrenal tissue is clearly dependent on the light–dark cycle, but it could also be regulated by feeding-related cues, at least when food is provided during the less-active (dark) phase [7,39]. The entrainment of the interrenal (homologous to mammalian adrenal) clock is especially interesting for endocrine function since a growing number of studies reveal the influence of this oscillator on glucocorticoid secretion in mammals [67,68]. In this direction, rhythms in adrenal clock genes and glucocorticoid secretion have even been observed in hypophysectomized rats [69,70].

Finally, one of the most striking results of the present work is the fact that animals do not need a fully functional interrenal clock to show daily cortisol rhythms (and locomotor activity rhythms). Contrary to FAA, general locomotor activity and daily cortisol rhythms seem to be driven by a LEO in goldfish. Proper functioning of the interrenal clock could be related to the sensitivity to ACTH, as previously suggested in mammals [71,72]. It could be hypothesized that rhythmic CRH and ACTH release (probably driven by LEOs in the hypothalamus) would evoke daily cortisol rhythms. However, proper functioning of the interrenal clock is required, as in the absence of *zeitgebers* (LD cycle or feeding time) the sensitivity of interrenal steroidogenic cells to ACTH seems to be altered.

## 5. Conclusions

All data considered, FAA in goldfish is not only the result of rhythmic clock genes in the HPI axis, because rhythmic clock gene expression was observed in randomly fed fish while FAA was not, and FAA was observed in fish that ate at a fixed time but had no clock gene expression rhythms in the axis (DD-2 group). The existence of FEOs in other locations that could be involved in maintaining FAA cannot be discarded. In this sense, clock genes are rhythmic in the liver and the gut of goldfish maintained under constant light [18,46] and constant darkness ([28], unpublished own data) with a scheduled feeding, supporting its function as FEOs in this species. Another less studied possibility would be that FAA is not an output directly generated by oscillations of the now identified as clock genes, but by a different still unknown circadian oscillator, since it is maintained in mutant mice with a disabled core clock [73].

The light-dark cycle plays a determinant role in the temporal homeostasis of the HPI axis in goldfish, while feeding time plays a secondary role. As a rule, the circadian oscillators in the hypothalamus, pituitary, and interrenal tissue could be considered as functional LEOs, although the interrenal tissue may also have some FEO characteristics. Disruptions of circadian functioning of HPI clocks caused by the removal of *zeitgebers* do not seem to affect FAA or the diurnal activity pattern, suggesting that FAA is not a direct output of these oscillators. However, such HPI clock alterations could impact cortisol day-night variations and drive an exacerbated response to ACTH.

The exact health impacts caused by circadian dysregulation in the HPI axis, while apparent, are still to be further investigated. On the same note, welfare strategies need to be implemented to reach an optimal acclimation of fish in the context of aquaculture.

## Data Availability

Data is contained within the article or supplementary material.

## Supplementary Materials

The following are available online at https://www.mdpi.com/2076-2615/11/3/669/s1, Figure S1: Polar representations of parameters defining clock gene rhythms, Table S1: Primers used in the RT-qPCR for housekeeping and target genes, Table S2: Parameters of the sinusoidal functions obtained by cosinor analysis for the expression of clock genes in hypothalamus, pituitary, and interrenal tissue of the goldfish.

## References

[1] Buhr E.D., Takahashi J.S. (2013). Molecular components of the mammalian circadian clock. Handb. Exp. Pharmacol..

[2] Cahill G.M. (2002). Clock mechanisms in zebrafish. Cell Tissue Res..

[3] Bell-Pedersen D., Cassone V.M., Earnest D.J., Golden S.S., Hardin P.E., Thomas T.L., Zoran M.J. (2005). Circadian rhythms from multiple oscillators: Lessons from diverse organisms. Nat. Rev. Genet..

[4] Albrecht U. (2012). Timing to Perfection: The Biology of Central and Peripheral Circadian Clocks. Neuron.

[5] Isorna E., de Pedro N., Valenciano A.I., Alonso-Gómez Á.L., Delgado M.J. (2017). Interplay between the endocrine and circadian systems in fishes. J. Endocrinol..

[6] Costa L.S., Serrano I., Sánchez-Vázquez F.J., López-Olmeda J.F. (2016). Circadian rhythms of clock gene expression in Nile tilapia (*Oreochromis niloticus*) central and peripheral tissues: Influence of different lighting and feeding conditions. J. Comp. Physiol. B Biochem. Syst. Environ. Physiol..

[7] Gómez-Boronat M., Sáiz N., Delgado M.J., de Pedro N., Isorna E. (2018). Time-Lag in Feeding Schedule Acts as a Stressor That Alters Circadian Oscillators in Goldfish. Front. Physiol..

[8] Damiola F., Le Minli N., Preitner N., Kornmann B., Fleury-Olela F., Schibler U. (2000). Restricted feeding uncouples circadian oscillators in peripheral tissues from the central pacemaker in the suprachiasmatic nucleus. Genes Dev..

[9] Sánchez-Vázquez F.J., Aranda A., Madrid J.A. (2001). Differential effects of meal size and food energy density on feeding entrainment in goldfish. J. Biol. Rhythms.

[10] Mistlberger R.E. (1994). Circadian food-anticipatory activity: Formal models and physiological mechanisms. Neurosci. Biobehav. Rev..

[11] Mendoza J., Challet E. (2009). Brain clocks: From the suprachiasmatic nuclei to a cerebral network. Neuroscientist.

[12] Stephan F.K. (2002). The “other” circadian system: Food as a Zeitgeber. J. Biol. Rhythms.

[13] Davidson A.J. (2006). Search for the feeding-entrainable circadian oscillator: A complex proposition. Am. J. Physiol. Regul. Integr. Comp. Physiol..

[14] Carneiro B.T.S., Araujo J.F. (2012). Food entrainment: Major and recent findings. Front. Behav. Neurosci..

[15] Clarke J.D., Coleman G.J. (1986). Persistent meal-associated rhythms in SCN-lesioned rats. Physiol. Behav..

[16] López-Olmeda J.F., Tartaglione E.V., De La Iglesia H.O., Sánchez-Vázquez F.J. (2010). Feeding entrainment of food-anticipatory activity and per1 expression in the brain and liver of zebrafish under different lighting and feeding conditions. Chronobiol. Int..

[17] Aranda A., Madrid J.A., Sánchez-Vázquez F.J. (2001). Influence of light on feeding anticipatory activity in goldfish. J. Biol. Rhythms.

[18] Feliciano A., Vivas Y., de Pedro N., Delgado M.J., Velarde E., Isorna E. (2011). Feeding time synchronizes clock gene rhythmic expression in brain and liver of goldfish (*Carassius auratus*). J. Biol. Rhythms.

[19] Sánchez-Vázquez F.J., Madrid J.A., Zamora S., Tabata M. (1997). Feeding entrainment of locomotor activity rhythms in the goldfish is mediated by a feeding-entrainable circadian oscillator. J. Comp. Physiol. A Sens. Neural Behav. Physiol..

[20] Schibler U., Gotic I., Saini C., Gos P., Curie T., Emmenegger Y., Sinturel F., Gosselin P., Gerber A., Fleury-Olela F. (2016). Clock-talk: Interactions between central and peripheral circadian oscillators in mammals. Cold Spring Harb. Symp. Quant. Biol..

[21] Dibner C., Schibler U., Albrecht U. (2010). The Mammalian Circadian Timing System: Organization and Coordination of Central and Peripheral Clocks. Annu. Rev. Physiol..

[22] Roenneberg T., Merrow M. (2003). The network of time: Understanding the molecular circadian system. Curr. Biol..

[23] Vatine G., Vallone D., Gothilf Y., Foulkes N.S. (2011). It’s time to swim! Zebrafish and the circadian clock. FEBS Lett..

[24] Sánchez-Bretaño A., Callejo M., Montero M., Alonso-Gómez Á.L., Delgado M.J., Isorna E. (2016). Performing a hepatic timing signal: Glucocorticoids induce gper1a and gper1b expression and repress gclock1a and gbmal1a in the liver of goldfish. J. Comp. Physiol. B Biochem. Syst. Environ. Physiol..

[25] Whitmore D., Foulkes N.S., Sassone-Corsi P. (2000). Light acts directly on organs and cells in culture to set the vertebrate circadian clock. Nature.

[26] Blanco A.M., Sundarrajan L., Bertucci J.I., Unniappan S. (2018). Why goldfish? Merits and challenges in employing goldfish as a model organism in comparative endocrinology research. Gen. Comp. Endocrinol..

[27] Azpeleta C., Sánchez-Bretaño A., Isorna E., Nisembaum L.G., Velarde E., De Pedro N., Alonso-Gómez A.L., Delgado M.J. (2011). Understanding the circadian system as a net of clocks: Daily expression of clock genes in the hypothalamus-pituitary-interrenal axis in *Carassius auratus*. Adv. Comp. Endocrinol..

[28] Nisembaum L.G., Velarde E., Tinoco A.B., Azpeleta C., De Pedro N., Alonso-Gómez A.L., Delgado M.J., Isorna E. (2012). Light-dark cycle and feeding time differentially entrains the gut molecular clock of the goldfish (*Carassius auratus*). Chronobiol. Int..

[29] Velarde E., Haque R., Iuvone P.M., Azpeleta C., Alonso-Gomez A.L., Delgado M.J. (2009). Circadian clock genes of goldfish, carassius auratus: CDNA cloning and rhythmic expression of period and cryptochrome transcripts in retina, liver, and gut. J. Biol. Rhythms.

[30] Wendelaar Bonga S.E. (1997). The stress response in fish. Physiol. Rev..

[31] Bernier N.J., Peter R.E. (2001). The hypothalamic-pituitary-interrenal axis and the control of food intake in teleost fish. Comp. Biochem. Physiol. B Biochem. Mol. Biol..

[32] Grassi Milano E., Basari F., Chimenti C. (1997). Adrenocortical and adrenomedullary homologs in eight species of adult and developing teleosts: Morphology, histology, and immunohistochemistry. Gen. Comp. Endocrinol..

[33] To T.T., Hahner S., Nica G., Rohr K.B., Hammerschmidt M., Winkler C., Allolio B. (2007). Pituitary-Interrenal Interaction in Zebrafish Interrenal Organ Development. Mol. Endocrinol..

[34] Mommsen T.P., Vijayan M.M., Moon T.W. (1999). Cortisol in teleosts: Dynamics, mechanisms of action, and metabolic regulation. Rev. Fish Biol. Fish..

[35] Spiga F., Walker J.J., Terry J.R., Lightman S.L. (2014). HPA axis-rhythms. Compr. Physiol..

[36] West A.C., Iversen M., Jørgensen E.H., Sandve S.R., Hazlerigg D.G., Wood S.H. (2020). Diversified regulation of circadian clock gene expression following whole genome duplication. PLoS Genet..

[37] Razzoli M., Karsten C., Yoder J.M., Bartolomucci A., Engeland W.C. (2014). Chronic subordination stress phase advances adrenal and anterior pituitary clock gene rhythms. Am. J. Physiol. Integr. Comp. Physiol..

[38] Ishida A., Mutoh T., Ueyama T., Bando H., Masubuchi S., Nakahara D., Tsujimoto G., Okamura H. (2005). Light activates the adrenal gland: Timing of gene expression and glucocorticoid release. Cell Metab..

[39] Girotti M., Weinberg M.S., Spencer R.L. (2009). Diurnal expression of functional and clock-related genes throughout the rat HPA axis: System-wide shifts in response to a restricted feeding schedule. Am. J. Physiol. Metab..

[40] Kalsbeek A., van der Spek R., Lei J., Endert E., Buijs R.M., Fliers E. (2012). Circadian rhythms in the hypothalamo-pituitary-adrenal (HPA) axis. Mol. Cell. Endocrinol..

[41] Azpeleta C., Martínez-Álvarez R.M., Delgado M.J., Isorna E., De Pedro N. (2010). Melatonin reduces locomotor activity and circulating cortisol in goldfish. Horm. Behav..

[42] Livak K.J., Schmittgen T.D. (2001). Analysis of relative gene expression data using real-time quantitative PCR and the 2-ΔΔCT method. Methods.

[43] Duggleby R.G. (1981). A nonlinear regression program for small computers. Anal. Biochem..

[44] Delgado M.J., Aeonso-Gómez A.L., Gancedo B., de Pedro N., Valenciano A.I., Alonso-Bedate M. (1993). Serotonin N-Acetyltransferase (NAT) Activity and Melatonin Levels in the Frog Retina Are Not Correlated during the Seasonal Cycle. Gen. Comp. Endocrinol..

[45] Challet E., Mendoza J., Dardente H., Pévet P. (2009). Neurogenetics of food anticipation. Eur. J. Neurosci..

[46] Sánchez-Vázquez F.J., Madrid J.A., Zamora S., Iigo M., Tabata M. (1996). Demand feeding and locomotor circadian rhythms in the goldfish, Carassius auratus: Dual and independent phasing. Physiol. Behav..

[47] Vera L.M., De Pedro N., Gómez-Milán E., Delgado M.J., Sánchez-Muros M.J., Madrid J.A., Sánchez-Vázquez F.J. (2007). Feeding entrainment of locomotor activity rhythms, digestive enzymes and neuroendocrine factors in goldfish. Physiol. Behav..

[48] Iigo M., Tabata M. (1996). Circadian rhythms of locomotor activity in the goldfish *Carassius auratus*. Physiol. Behav..

[49] López-Olmeda J.F., Madrid J.A., Sánchez-Vázquez F.J. (2006). Melatonin effects on food intake and activity rhythms in two fish species with different activity patterns: Diurnal (goldfish) and nocturnal (tench). Comp. Biochem. Physiol. A Mol. Integr. Physiol..

[50] Vera L.M., Negrini P., Zagatti C., Frigato E., Sánchez-Vázquez F.J., Bertolucci C. (2013). Light and feeding entrainment of the molecular circadian clock in a marine teleost (*Sparus aurata*). Chronobiol. Int..

[51] Sánchez J.A., López-Olmeda J.F., Blanco-Vives B., Sánchez-Vázquez F.J. (2009). Effects of feeding schedule on locomotor activity rhythms and stress response in sea bream. Physiol. Behav..

[52] Ceinos R.M., Chivite M., López-Patiño M.A., Naderi F., Soengas J.L., Foulkes N.S., Míguez J.M. (2019). Differential circadian and light-driven rhythmicity of clock gene expression and behaviour in the turbot, *Scophthalmus maximus*. PLoS ONE.

[53] Cavallari N., Frigato E., Vallone D., Fröhlich N., Lopez-Olmeda J.F., Foà A., Berti R., Sánchez-Vázquez F.J., Bertolucci C., Foulkes N.S. (2011). A Blind Circadian Clock in Cavefish Reveals that Opsins Mediate Peripheral Clock Photoreception. PLoS Biol..

[54] Oike H., Oishi K., Kobori M. (2014). Nutrients, Clock Genes, and Chrononutrition. Curr. Nutr. Rep..

[55] Oster H., Challet E., Ott V., Arvat E., de Kloet E.R., Dijk D.J., Lightman S., Vgontzas A., Van Cauter E. (2017). The functional and clinical significance of the 24-h rhythm of circulating glucocorticoids. Endocr. Rev..

[56] Tahara Y., Aoyama S., Shibata S. (2017). The mammalian circadian clock and its entrainment by stress and exercise. J. Physiol. Sci..

[57] Helfrich-Förster C., Albrecht U., Pilorz V., Zhao J., Chen L., Xie Y., Tang Q., Chen G., Xie M., Yu S. (2019). New Insights Into the Circadian Rhythm and Its Related Diseases. Front. Physiol..

[58] Montoya A., López-Olmeda J.F., Garayzar A.B.S., Sánchez-Vázquez F.J. (2010). Synchronization of daily rhythms of locomotor activity and plasma glucose, cortisol and thyroid hormones to feeding in Gilthead seabream (*Sparus aurata*) under a light-dark cycle. Physiol. Behav..

[59] Minnetti M., Hasenmajer V., Pofi R., Venneri M.A., Alexandraki K.I., Isidori A.M. (2020). Fixing the broken clock in adrenal disorders: Focus on glucocorticoids and chronotherapy. J. Endocrinol..

[60] Lazado C.C., Pedersen P.B., Nguyen H.Q., Lund I. (2017). Rhythmicity and plasticity of digestive physiology in a euryhaline teleost fish, permit (*Trachinotus falcatus*). Comp. Biochem. Physiol. Part A Mol. Integr. Physiol..

[61] Patiño M.A.L., Rodríguez-Illamola A., Conde-Sieira M., Soengas J.L., Míguez J.M. (2011). Daily rhythmic expression patterns of clock1a, bmal1, and per1 genes in retina and hypothalamus of the rainbow trout, oncorhynchus mykiss. Chronobiol. Int..

[62] Karolczak M., Burbach G.J., Sties G., Korf H.-W., Stehle J.H. (2004). Clock gene mRNA and protein rhythms in the pineal gland of mice. Eur. J. Neurosci..

[63] Asher G., Gatfield D., Stratmann M., Reinke H., Dibner C., Kreppel F., Mostoslavsky R., Alt F.W., Schibler U. (2008). SIRT1 Regulates circadian clock gene expression through PER2 deacetylation. Cell.

[64] Gooley J.J., Schomer A., Saper C.B. (2006). The dorsomedial hypothalamic nucleus is critical for the expression of food-entrainable circadian rhythms. Nat. Neurosci..

[65] Mieda M., Williams S.C., Richardson J.A., Tanaka K., Yanagisawa M. (2006). The dorsomedial hypothalamic nucleus as a putative food-entrainable circadian pacemaker. Proc. Natl. Acad. Sci. USA.

[66] Fuller P.M., Lu J., Saper C.B. (2008). Differential rescue of light- and food-entrainable circadian rhythms. Science.

[67] Son G.H., Chung S., Choe H.K., Kim H.-D., Baik S.-M., Lee H., Lee H.-W., Choi S., Sun W., Kim H. (2008). Adrenal peripheral clock controls the autonomous circadian rhythm of glucocorticoid by causing rhythmic steroid production. Proc. Natl. Acad. Sci. USA.

[68] Oster H., Damerow S., Kiessling S., Jakubcakova V., Abraham D., Tian J., Hoffmann M.W., Eichele G. (2006). The circadian rhythm of glucocorticoids is regulated by a gating mechanism residing in the adrenal cortical clock. Cell Metab..

[69] Fahrenkrug J., Hannibal J., Georg B. (2008). Diurnal rhythmicity of the canonical clock genes Per1, Per2 and Bmal1 in the rat adrenal gland is unaltered after hypophysectomy. J. Neuroendocrinol..

[70] Meier A.H. (1976). Daily variation in concentration of plasma corticosteroid in hypophysectomized rats. Endocrinology.

[71] Torres-Farfan C., Abarzua-Catalan L., Valenzuela F.J., Mendez N., Richter H.G., Valenzuela G.J., Serón-Ferré M. (2009). Cryptochrome 2 expression level is critical for adrenocorticotropin stimulation of cortisol production in the capuchin monkey adrenal. Endocrinology.

[72] Richter H.G., Torres-Farfan C., Garcia-Sesnich J., Abarzua-Catalan L., Henriquez M.G., Alvarez-Felmer M., Gaete F., Rehren G.E., Seron-Ferre M. (2008). Rhythmic expression of functional MT1 melatonin receptors in the rat adrenal gland. Endocrinology.

[73] Storch K.F., Weitz C.J. (2009). Daily rhythms of food-anticipatory behavioral activity do not require the known circadian clock. Proc. Natl. Acad. Sci. USA.

