# Supplementary material for: The Lack of Light-Dark and Feeding-Fasting Cycles Alters Temporal Events in the Goldfish (Carassius auratus) Stress Axis
Source: Animals (Basel). 2021 Mar 3;11(3):669. doi: 10.3390/ani11030669 (PMC7998219; doi:10.3390/ani11030669)
Supplement: Supplementary file 1 [file animals-11-00669-s001.pdf]

animals

MDPI

Table S1. Primers used in the RT-qPCR for housekeeping and target genes.

Gene	Access number (genbank)	Primer sequences		Product (bp)
β-actin	AB039726.2	Forward	CGGGAGTGATGGTTGGCA	168
		Reverse	AACACGCAGCTGTTGTAGA	
bmal1a	KF840401.1	Forward	GATTCTGTTCGTCTCGGAG	161
		Reverse	ATCGATGAGTCTTCCCGTG	
clock1a	KJ574204.1	Forward	CGATGGCAGCATCTCTTGTGT	187
		Reverse	TCCTGGATCTGCCGCAGTTCAT	
per1a	EF690698.1	Forward	CAGTGGCTCGAATGAGCACCA	155
		Reverse	TGAAGACCTGCTGTCCGTTGG	
per1b	KP663726.1	Forward	CTCGCAGCTCCACAAACCTA	235
		Reverse	TGATCGTGCAGAAGGAGCCG	

Table S2: Parameters of the sinusoidal functions obtained by COSINOR analysis for the expression of clock genes in hypothalamus, pituitary and interrenal tissue of the goldfish (mean ± SEM).

		Hypothalamus			Pituitary			Interrenal tissue		
		Mesor (fold change)	Amplitude (fold change)	**Φ Acrophase (ZT/CT h)**	Mesor (fold change)	Amplitude (fold change)	**Φ Acrophase (ZT/CT h)**	Mesor (fold change)	Amplitude (fold change)	**Φ Acrophase (ZT/CT h)**
per1a	**LD-2**	9.52 ± 0.9	10.5 ± 1.2	**22.3 ± 0.4**	20.5 ± 2.8	26.16 ± 4.1	**22.01 ± 0.5**	11.09 ± 2	8.86 ± 2.9	**22.1 ± 1.1**
	**LD-R**	5.39 ± 0.5	4.49 ± 0.8	**21.9 ± 0.6**	10.18 ± 1.3	11.57 ± 1.9	**21.79 ± 0.6**	8.04 ± 1.2	7.53 ± 1.6	**22.6 ± 0.8**
	**DD-2**	-	-	**-**	-	-	**-**	-	-	**-**
per1b	**LD-2**	8.98 ± 1	9.23 ± 1.4	**23.1 ± 0.6**	9.41 ± 1.3	10.04 ± 1.8	**23.02 ± 0.7**	4.39 ± 0.6	5.05 ± 0.9	**0.06 ± 0.6**
	**LD-R**	6.7 ± 0.9	6.72 ± 1.3	**23.4 ± 0.7**	8.65 ± 1.5	10.25 ± 2.2	**22.42 ± 0.8**	9.79 ± 0.8	11.01 ± 1.5	**23.47 ± 1.2**
	**DD-2**	-	-	**-**	-	-	**-**	-	-	**-**
clock1a	**LD-2**	1.77 ± 0.1	0.57 ± 0.1	**13.1 ± 0.6**	2.36 ± 0.2	1.84 ± 0.3	**11.98 ± 0.5**	2.10 ±0.1	1.27 ± 0.2	**13.09 ± 0.6**
	**LD-R**	-	-	**-**	2.85 ± 0.2	1.78 ± 0.3	**10.43 ± 0.7**	-	-	**-**
	**DD-2**	2.01 ± 0.2	1.17 ± 0.3	**6.88 ± 0.9**	-	-	**-**	-	-	**-**
bmal1a	**LD-2**	4.4 ± 0.2	2.67 ± 0.2	**8.83 ± 0.3**	4.39 ± 0.4	3.32 ± 0.6	**9.89 ± 0.6**	2.80 ± 0.3	1.69 ± 0.5	**10.80 ± 1**
	**LD-R**	2.51 ± 0.2	1.28 ± 0.3	**8.01 ± 0.9**	3.03 ± 0.3	2.09 ± 0.4	**10.57 ± 0.7**	-	-	-
	**DD-2**	-	-	**-**	-	-	-	-	-	-

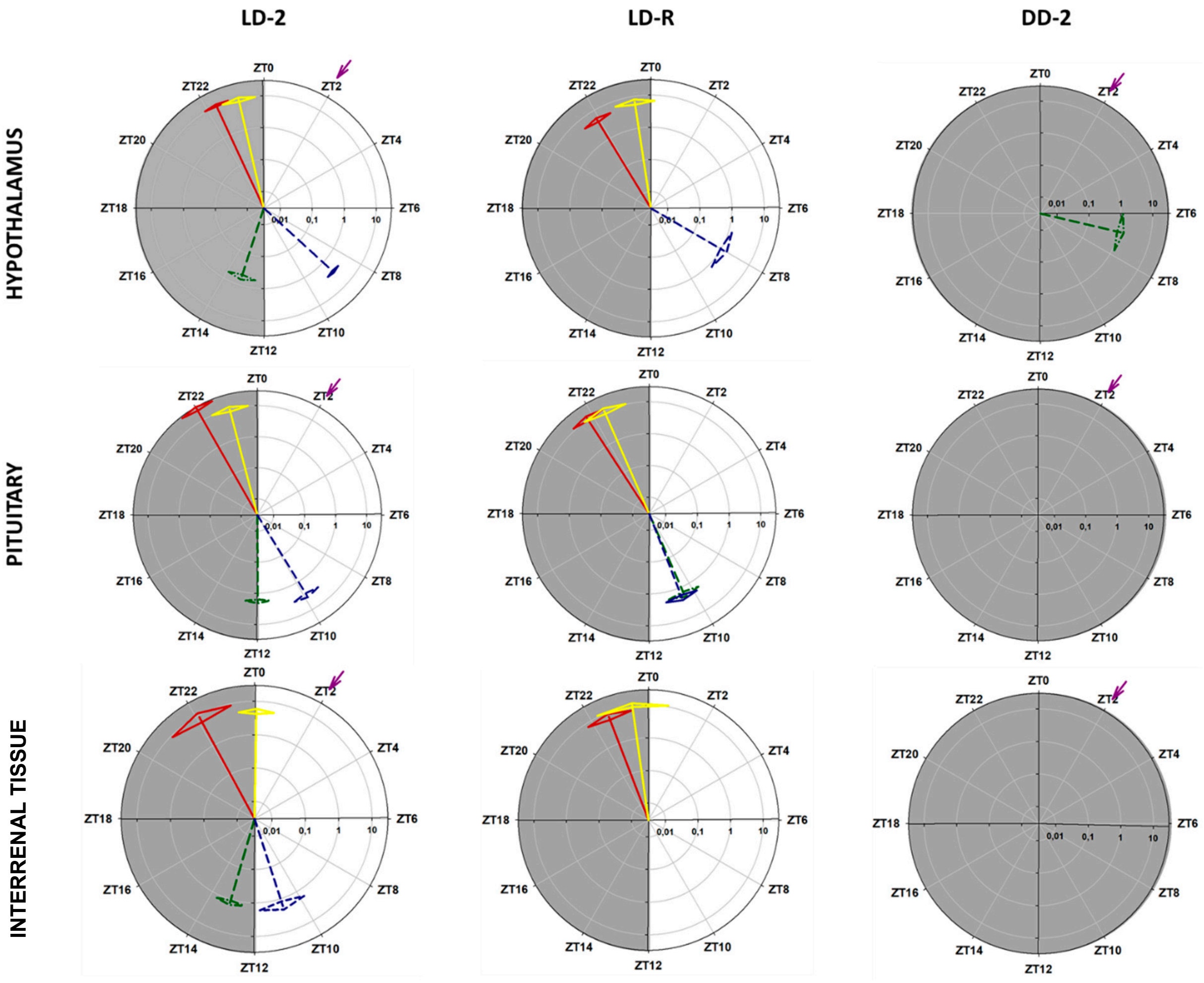

Figure S1: Polar representations of parameters defining clock gene rhythms: *per1a* (red), *per1b* (yellow), *clock1a* (green), *bmal1a* (blue). The length of the vector (radial axis) indicates the value of the amplitude (fold change of relative expression in logarithmic scale). The angular position indicates the acrophase (ZT, *zeitgeber* time). The grey areas indicate the darkness period and purple arrow points to the feeding time when it was fixed. The SEM of these two parameters is represented by the rhombus at the end of each vector.
